# Decrease in the incidence of total hip arthroplasties in patients with rheumatoid arthritis - results from a well defined population in south Sweden

**DOI:** 10.1186/ar3328

**Published:** 2011-04-21

**Authors:** Korosh Hekmat, Lennart Jacobsson, Jan-Åke Nilsson, Ingemar F Petersson, Otto Robertsson, Göran Garellick, Carl Turesson

**Affiliations:** 1 Section of Rheumatology, Department of Clinical Sciences, Malmö, Lund University and Skåne University Hospital, Södra Förstadsgatan 101, 205 02 Malmö, Sweden; 2 Orthopedics, Department of Clinical Sciences Lund, Lund University and Skåne University Hospital, Södra Förstadsgatan 101, 205 02 Malmö, Sweden; 3 Department of Orthopaedics, Swedish Hip Arthroplasty Register, Institute of Clinical Sciences at Sahlgrenska Academy, University of Gothenburg and Sahlgrenska University Hospital, Blå Stråket 5, 413 45 Gothenburg, Sweden

## Abstract

**Introduction:**

One aim of modern pharmacologic treatment in rheumatoid arthritis (RA) is to prevent joint destruction and reduce the need for surgery. Our purpose was to investigate secular trends in the incidence of primary total hip and knee arthroplasties in a well defined sample of patients with RA.

**Methods:**

Prevalent cases with RA in 1997 and incident cases from 1997 to 2007 in a community based register in Malmö, south Sweden, were included. Based on a structured review of the medical records, patients were classified according to the 1987 ACR criteria for RA. This cohort was linked to the Swedish Hip Arthroplasty Register (through December 2006) and the Swedish Knee Arthroplasty Register (through October 2007). Patients with a registered total hip or knee arthroplasty before 1997 or before RA diagnosis were excluded. Incidence rates for the period of introduction of TNF inhibitors (1998 to 2001) were compared to the period when biologics were part of the established treatment for severe RA (2002 to 2006/2007).

**Results:**

In the cohort (*n *= 2,164; 71% women) a primary hip arthroplasty was registered for 115 patients and a primary knee arthroplasty for 82 patients. The incidence of primary total hip arthroplasties decreased from the period 1998 to 2001 (12.6/1,000 person-years (pyr)) to 2002 to 2006 (6.6/1,000 pyr) (rate ratio (RR) 0.52; 95% confidence interval (CI) 0.35 to 0.76). There was a trend towards an increase of primary knee arthroplasties (incidence 4.8/1,000 pyr vs. 6.8/1,000 pyr; RR 1.43; 95% CI 0.89 to 2.31).

**Conclusions:**

Our investigation shows a significant decrease in the incidence of total hip arthroplasties in patients with RA after 2001. Possible explanations include a positive effect on joint damage from more aggressive pharmacological treatment.

## Introduction

Rheumatoid arthritis (RA) is a systemic inflammatory disease in which persistent active inflammation leads to major joint destruction. Chronic destructive arthritis causes suffering and impaired function for the patient as well as substantial costs for the health care system and society due to increased need for hospital admissions and orthopedic surgery. RA has also been associated with increased mortality compared with the general population [1], and increased incidence of cardiovascular disease in patients with RA has been confirmed in studies in recent years [2,3].

The aim of pharmacological treatment in RA is to reduce inflammation, improve function and prevent long-term joint damage. Disease-modifying anti-rheumatic drugs (DMARDs), including inhibitors of tumor necrosis factor (TNF) and other biologic immunomodulating agents, are used to reduce inflammation and disease progression. These medications are efficient in many patients, but are also costly.

Health economic evaluations of the treatment for RA need to take into account the impact of indirect costs (for example, from sick leave or early retirement) and direct medical costs (health care resource utilization, including admissions and surgery). Orthopedic surgery has been increasingly available in recent years and total joint arthroplasty is used earlier and more frequently in osteoarthritis. A recent study suggests that this has not been the case in patients with RA, possibly due to successful early treatment of inflammation [4]. Two other studies have shown an overall reduced rate over time of joint surgery in patients with RA [5,6].

In a study of a population based RA sample from Rochester, Minnesota, the cumulative incidence of orthopedic surgery by decade of RA diagnosis was investigated. In this sample, patients diagnosed with RA after 1985 were less likely to require joint surgery overall [5]. A study performed at the Department of Orthopaedic Surgery, Karolinska University Hospital, Stockholm, Sweden, showed a decrease in the rates of hospital admission caused by lower limb surgery in patients with rheumatoid arthritis between 1987 and 2001. This may reflect trends in disease severity, management, and health outcomes of this disease in Sweden [6]. Issues such as the impact of differences in access to health care and changes in the management of RA on the need for orthopedic surgery in patients should be further studied.

The Swedish national registers for hip and knee arthroplasties are excellent resources for the study of changes in the incidence of such procedures over time. Previous analyses based on the Swedish Hip Arthroplasty Register demonstrated a decline in the proportion of total hip arthroplasties (THA) due to inflammatory joint disease from 5% of all THA during the period 1992 to 2002 to 2% in 2007 [7]. A recent survey from the Swedish Knee Arthroplasty Register showed that the incidence of total knee arthroplasties (TKA) in Sweden with a diagnosis of RA noted in the register declined during the period 1997 to 2007 from 6/100,000 to 2/100,000 [8]. Although these studies provide reliable information on THA and TKA, the RA diagnoses used have not been validated, and the denominator population of patients with RA is not defined.

The aim of the present study was to investigate trends in the incidence of primary THA and TKA in a well defined sample of patients with RA. In order to do this we studied the incidence of first hip and knee joint arthroplasty in a community setting, using a register containing the majority of patients with RA in this geographical area.

## Materials and methods

### Patients with RA

In 1997, a register of all known patients with RA in the city of Malmö, Sweden, was established. Inclusion was based on a clinical diagnosis of RA by a rheumatologist and fulfilment of the 1987 American College of Rheumatology (ACR) criteria for RA [9].

Patients were recruited from the rheumatology outpatient clinic of Malmö University Hospital, which is the only hospital serving the city, and from the four rheumatologists in private practice in Malmö [10]. The prevalence of RA and the sex and age distributions in the Malmö RA register were found to be comparable to the RA prevalence in studies from Halland in Sweden, and to the data from a population-based RA register in Oslo, Norway [11,12]. Subsequent surveys using the diagnostic index of primary care centers and questionnaires sent to other physicians in the area indicate that >90% of all patients with diagnosed RA in the city at that time were included in the register. All patients are registered using the unique national 10-digit ID number assigned to all Swedish residents who were alive in 1947 or born thereafter. Additional patients with a registered diagnosis of RA were identified using the Swedish National Patient Register [13] and the local patient administrative system, and after 2002 a continuously updated register was established including previously identified patients and new cases of RA seen by a rheumatologist in Malmö. The register has previously been used for studies of RA related co-morbidities [2,14,15]. Patients identified through 2006 were included in the present study.

For a major proportion, 1,918 of the total of 2,419 patients known to the register, the year of RA diagnosis had been reported by the managing rheumatologist, or the patients had been classified in a previous review which was part of another research study [16,17]. For the remaining 501 patients, a structured review of the medical records was performed. The patients were classified according to the 1987 ACR criteria and the year of diagnosis was noted. This led to the exclusion of 133 cases, for which other diagnoses were considered more relevant and the 1987 ACR criteria were not fulfilled. For an additional 72 cases, there were insufficient data in the medical records to support a diagnosis of RA. A total of 50 cases had a diagnosis of RA before age 16. These were also excluded. A total of 2,164 patients were, therefore, included in the present study.

Data on vital status were retrieved up to 31 December 2007 from the Swedish Causes of Death Register. The Causes of Death Register is administered by the National Board of Health and Welfare. In 1996, the register was estimated to include data on 99.42% of all deaths [18].

### Hip and knee arthroplasty registers

The number of performed THAs and TKAs was obtained from the Swedish Hip and Knee Arthroplasty registries. The Swedish Hip Arthroplasty register was established in 1979, in Gothenburg, and includes data on 98 to 99% of THAs performed in Sweden. Individual patient data were available from January 1992. All 80 public and private hospitals in Sweden that perform these operations participate in the Register. The steering group of the Swedish Hip Arthroplasty Register also analyses many different outcome aspects of THA surgery and provides feedback to the profession. For example, an improvement in prosthesis survival over time has been reported [7].

The Swedish Knee Arthroplasty Register was established in 1975 and includes data on estimated 96% of TKA performed in Sweden. All 79 units who perform knee arthroplasty keep providing information to the Register. The register provides important prosthesis survival feedback in both RA and osteoarthritis [19]. Individual patient data were available from the establishment of the register in 1975.

These registers were linked to the Malmö RA register in order to identify first knee and hip arthroplasty procedures in patients with RA. Data on primary THA performed in Sweden were available until December 2006 and on primary TKA until October 2007. Patients with a registered hip or knee arthroplasty before the start of the study period in 1997 (when the RA register was established, before which the denominator can not be defined), or before RA diagnosis, were excluded. Subjects were censored at death, migration from Sweden, the first hip or knee arthroplasty, respectively, or at the close of the study.

### Statistical analysis

The total follow-up (number of person-years at risk) was calculated for each calendar year from 1998 through 2007, and the annual incidence rate of first THA and first TKA was estimated. The incidence rate for the period 1998 to 2001 was compared to that of 2002 to 2006 (for THA) or 2002 to 2007 (for TKA). Using the Poisson distribution ratio, 95% confidence intervals (CI) for incidence rates and incidence rate ratios were estimated.

All patients gave their informed consent to be included in the Malmö RA register and the Swedish hip and knee arthroplasty registers. No informed consent was obtained specifically for the present study. This procedure, and the study protocol, was approved by the Regional Ethical Review Board in Lund, Sweden.

## Results

**Figure 1:**
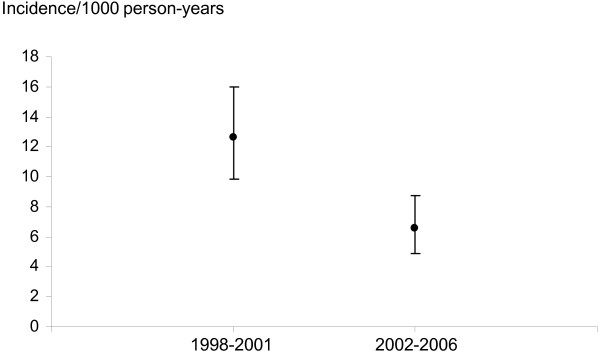
**Incidence of THA by study subperiod**. Estimated incidence of first primary total hip arthroplasty in 1998 to 2001 vs 2002 to 2006 (95% confidence interval).

**Figure 2:**
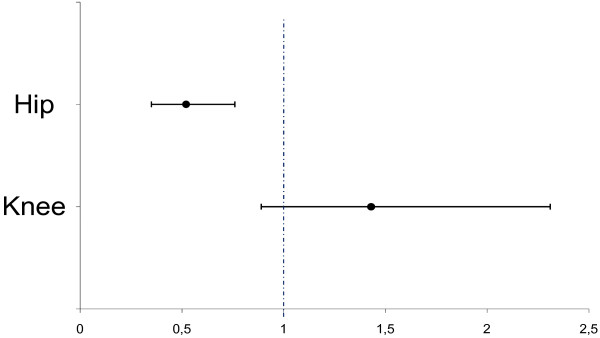
**Incidence rate ratios for THA and TKA in the second and first study subperiods**. Incidence rate ratio for first primary total hip and knee arthroplasty for 2002 to 2006/2007 vs 1998 to 2001 (95% confidence interval).

**Figure 3:**
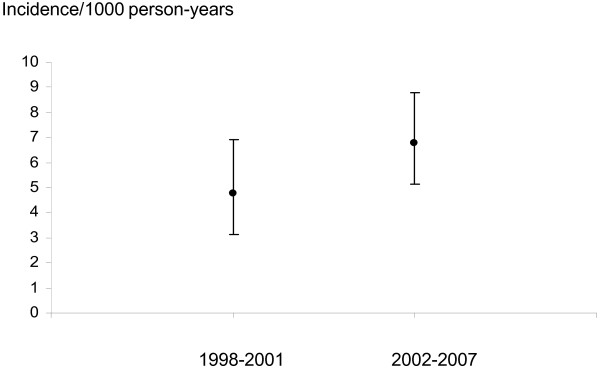
**Incidence of TKA by study subperiod**. Estimated incidence of first primary total knee arthroplasty in 1998 to 2001 vs 2002 to 2007(95% confidence interval).

A community-based sample of 2,164 patients with a validated diagnosis of RA according to the 1987 ACR criteria was studied (Table 1). Among these, 1,545 were women (71.4%). Mean age at diagnosis was 51 years (standard deviation 16.7, range 16 to 90). A total of 110 cases with a registered THA and 123 cases with a registered TKA before the study period were excluded. During the study period, there were 115 primary THA performed (Table 2) and 82 patients had a primary TKA (Table 3). Sixty-nine hip (12.6/1,000 person-years) and 27 knee arthroplasties (4.8/1,000 person-years) were performed between 1998 and 2001. Between 2002 and 2006, 46 THA were performed, corresponding to a lower incidence compared to the proceeding period (6.6/1,000 person-years; rate ratio (RR) 0.52; 95% confidence interval (CI) 0.35 to 0.76 for 2002 to 2006 vs.1998 to 2001) (Figures 1 and 2). The incidence of knee arthroplasty was slightly higher in 2002 to 2007 compared to the preceding period (*n *= 55; 6.8/1,000 person-years RR 1.43; 95% CI 0.89 to 2.31 for 2002 to 2007 vs 1998 to 2001) (Figures 2 and 3). The mean age at the start of each year of the studied cohort increased slightly over time (from 60.2 years in 1998 to 61.7 years in the THA analysis (Table 2), and from 60.6 years in 1998 to 62.9 years in 2007 in the TKA analysis (Table 3)).

**Table 1:** Characteristics of all included RA patients

N	2,164
Female	1,545 (71%)
Mean age at RA diagnosis (SD)	51.4 (16.7)
RA diagnosis in 1997 or earlier	1,481 (68.4%)
RA diagnosis after 1997	683 (31.6%)

**Table 2:** Incidence of primary THA during the study period

Year	Mean age*	Primary THA (n)	Person-years (pyr)	Incidence/1,000 pyr	95% CI
1998	60.2	13	1,341	9.69	5.16 to 16.58
1999	60.5	21	1,361	15.43	9.55 to 23.59
2000	60.9	16	1,381	11.59	6.62 to 18.81
2001	61.0	19	1,398	13.59	8.18 to 21.22
2002	61.0	10	1,397	7.16	3.43 to 13.16
2003	61.1	13	1,431	9.08	4.84 to 15.53
2004	61.2	5	1,461	3.42	1.11 to 7.99
2005	61.2	6	1,490	4.03	1.48 to 8.76
2006	61.7	12	1,467	8.18	4.23 to 14.29

THA, total hip arthroplasty. * Mean age for all observed patients in the cohort at the start of each calendar year

**Table 3:** Incidence of primary TKA during the study period

Year	Mean age*	Primary knee TJR (n)	Person-years (pyr)	Incidence/1000 pyr	95% CI
1998	60.6	9	1,369	6.57	3.01 to 12.48
1999	61.0	5	1,395	3.58	1.16 to 8.36
2000	61.5	6	1,423	4.22	1.55 to 9.18
2001	61.6	7	1,439	4.86	1.96 to 10.02
2002	61.6	4	1,442	2.77	0.76 to 7.10
2003	61.7	14	1,463	9.57	5.23 to 16.06
2004	61.7	10	1,480	6.76	3.24 to 12.43
2005	61.6	12	1,491	8.05	4.16 to 14.06
2006	62.2	6	1,467	4.09	1.50 to 8.90
2007	62.9	9	1,062	8.47	3.88 to 16.09

TKA, total knee arthroplasty. * Mean age for all observed patients in the cohort at the start of each calendar year

## Discussion

In this study of a well defined RA population, we demonstrated a reduced incidence of primary THA in 2002 to 2006 compared to 1998 to 2001. By contrast, there was no significant change in the incidence of TKA. Our results are compatible with previous studies indicating a reduced rate of joint surgery overall in patients with RA [5,6], and a decreased rate of arthroplasty relative to the non-RA background population [4,7]. In contrast with our findings, the California State Database reported a decrease over time in 1983 to 2001 in the rate of primary TKA in patients with RA [20]. Changes in coding practice and reimbursement for joint replacement surgery may explain the results from the latter study, although we cannot exclude that a global decline in the frequency of knee arthroplasties has been followed by stabilization or increase after the turn of the century.

Our results also differ from recent data from a Scandinavian survey of the national knee arthroplasty registers, in which a reduced incidence of TKA in Sweden over time was found [8]. Several methodological issues may explain these discrepancies. First, the present study used a defined set of RA patients with a validated diagnosis, whereas in the national TKA study the diagnosis of RA was based on the report from the orthopedic surgeon. Increasing misclassification of overall milder cases of RA over time would affect analyses of longitudinal trends. Second, we studied the incidence of the first TKA in the local RA population; whereas the national study investigated the incidence of all RA related TKA, with the entire Swedish population as the denominator. In the national study, a systematic change in the reporting of RA patients to the register may bias the findings. On the other hand, the precision of the estimates in the present study are limited due to the small sample size. Finally, there was no decrease in the incidence of RA related TKA in Denmark in the Scandinavian survey, indicating that geographical differences may play a role.

Potential explanations for the reduced rate of THA include reduced RA related joint damage due to better management of RA. Treatment strategies for RA have changed markedly over the past three decades, with the introduction of early and aggressive treatment. In the present study population an increasing use of DMARDs (52% to 87%) and TNF-inhibitors (0% to 20%) from 1997 to 2005 together with substantial improvements in median health assessment questionnaire disability index and Short Form (36) health survey score levels have previously been reported [21]. The timing of our study thus coincides with the establishment of tumor necrosis factor (TNF) inhibitors in the standard of care of patients with severe RA. These agents were introduced in the late 1990's, and used more extensively after 2002. There is extensive evidence for a reduced peripheral joint damage in patients with RA treated with TNF inhibitors [22,23], and such treatment could also prevent hip destruction. Other changes in RA management may also have contributed to the decline in THA surgery.

The contrasting pattern for knee arthroplasties may indicate that mechanisms of joint destruction may be partly different in knees and hips. In a systematic study of multiple sections from the cartilage-pannus junction of RA joints, invasive pannus formation with major cartilage degradation was more frequent in hip joints, and osteophyte formation was more frequent in knee joints [24]. TNF inhibitors and other drugs could, therefore, be less successful in preventing knee damage. A second possible explanation could be that criteria for knee arthroplasty in RA have changed over time or TKA has become more available compared to THA. Such hypothetical changes could possibly result in TKA being performed in patients with less severe disease, relative to THA. However, there are presently no data supporting such changes in Sweden.

Limitations of the present study are due to the sample size, which results in a limited number of primary joint arthroplasties. In addition, the patients were mostly Caucasians from a single urban area in southern Sweden, and the findings may not apply to other settings. In theory, the results could partly reflect local changes in this district, but we consider it unlikely that changes in indications for surgery would be substantially different from other areas in Scandinavia. Our study is based on only eight years of observation, but the need for total joint arthroplasty is an important severe long-term outcome of RA and should be investigated over longer periods. On the other hand, some long term studies have suffered from problems related to changes in coding practice (such as the transition from the International Classification of Diseases (ICD) -9 to the ICD-10 system in 1998 for a previous national Swedish study) [6].

The mean age of the cohort increased slightly over time. Based on this, a minor increase in the rate of incident arthroplasties would be expected, but this is unlikely to have had any major impact on our results. Changes in body mass index and physical activity may also have influenced our results, but no such data are available.

Major strengths of our study include the community based approach, which limits selection bias, and the validated RA diagnosis based on the 1987 ACR criteria, which contrasts with studies based on patient administrative databases alone. Furthermore, the use of national registers for joint arthroplasties mean that we were likely to identify virtually all THA and TKA in the cohort, including those performed in hospitals in other parts of Sweden.

## Conclusions

We have demonstrated a reduced rate of THA surgery, but not TKA, over time in patients with RA. Our observation reflects only indirect evidence of reduced joint damage in rheumatoid arthritis. Further studies are needed to follow changes in the need of arthroplasties in RA, and to investigate the underlying mechanisms.

## Abbreviations

ACR: American College of Rheumatology; CI: confidence interval; DMARDs: disease-modifying anti-rheumatic drugs; ICD: International Classification of Diseases; pyr: person-years; RA: rheumatoid arthritis; RR: rate ratio; THA: total hip arthroplasties; TKA: total knee arthroplaties; TNF: tumor necrosis factor

## Competing interests

The authors declare that they have no competing interests.

## Authors' contributions

KH performed the medical record review, participated in the design of the study and the statistical analysis, and drafted the manuscript. LJ participated in the design of the study and in the analysis and interpretation of data. J-ÅN participated in the design of the study and performed the statistical analysis. IFP participated in the design of the study and in the analysis and interpretation of data. OR and GG contributed data from the knee and hip arthroplasty registers and participated in the analysis and interpretation of data. CT conceived of the study, assisted in the medical record review, participated in the statistical analysis and helped draft the manuscript. All authors read and approved the final manuscript.
